# The Hospital World

**Published:** 1910-12-24

**Authors:** 


### The Hospital World.

### A MUNIFICENT FRIEND TO THE HOSPITALS.

### The Late Mr. Henry Silver.

On December 3 last there died, at his residence in Princes Gardens, S.W., an old gentleman who for more than half a century had taken a quiet but active interest in the work of the voluntary hospitals of this country, and who showed his practical sympathy with such work by leaving to three of the best-known London institutions very large legacies in his will. Leaving property which is valued at nearly £1,200,000, he directed that a sum of £50,000 should be paid by his executors to the Royal Free Hospital, Gray's Inn Road, W.C.; a sum of £25,000 to the Victoria Hospital for Children, Chelsea, and a similar sum of £25,000 to the Hospital for Sick Children, Great Ormond Street, W.C., together with further smaller bequests, which include a life annuity of £500 to his nurse.

To the public Mr. Henry Silver, by whose will these charities so largely benefit, was almost unknown. An older generation knew him as a writer on the staff of *Punch*, where his pen was always at the service of charity, and those who had the privilege of his personal friendship were aware of the fact that he took a deep, and in some respects a very active, interest in hospital work; but it is safe to say that even his intimates hardly appreciated the depth of that interest. Born in 1828, Mr. Silver early in life attended Charterhouse School, where he had as one of his classmates William Makepeace Thackeray, the novelist, who was afterwards his colleague. Subsequently Mr. Silver took up literary work, and contributed to various London papers. Even before he had attained his majority he wrote for *Punch*, and ten years after his first contribution had been accepted he joined the staff of that paper, and was a regular attendant at the weekly table in Messrs. Agnew's establishment in Bouverie Street. In his own circle he was well known and esteemed for his geniality and kindliness, though the fact that he was such a rich man as his will has shown was not even guessed by his contemporaries. In 1870 he retired from active service as a journalist, and devoted himself largely to private charity work. His leisure and his wealth permitted him to take an interest in many benevolent societies, and he took care to become better acquainted with institutions, such as the Royal Free and the children's hospitals, on whose behalf he had employed his pen while on the staff of the London Charivari. Shortly after his retirement from the *Punch* staff he was elected to the Board of Management of the Royal Free Hospital. He took his duties seriously and devoted himself whole-heartedly to the work, rarely missing a Board meeting for thirty-six years, though of late, since the death of his wife two years ago, his impaired health forced him to give up much of his active hospital work. He gave willingly towards its fund, and proved himself as generous a supporter as before his election to the Board he had been a kind friend to the institution, which still makes capital out of the notice he contributed to the columns of *Punch* many years ago. "The Royal Free Hospital," he wrote, "is not more free than welcome as a charitable helping-place to thousands of our poor. When first it was started, not a hospital in London was ever freely open, as in charity all should be, to such sick folk as the Royal Free was founded specially to succour. In this really useful hospital, so long as there are funds unspent and sleeping wards unfilled, any sick poor persons may come to them and fill them; and they need not lose their little strength by hunting up subscribers to send them 'Open Sesame' in an admission ticket." The secretary of the institution has taken care that this excellent tribute to the merits of the hospital shall not be forgotten by displaying it prominently on the leaflets, which set forth the work that the Royal Free is doing for the sick poor of its district and of London generally. Mr. Silver's interest was no doubt specially roused by this institution, but although he was not a member of the governing bodies of either of the two children's hospitals which he has so generously remembered in his will, he was nevertheless profoundly interested in their work, and took care to assure himself that they were in every way worthy of support. It is well worth bearing in mind that his will was signed only two days before his death, and that the legacy of £25,000 to the Great Ormond Street Hospital was therefore deliberately given at a time when this institution had been made the subject of an organised attack on the part of a certain sensation-mongering lay weekly. Mr. Silver's bequest may therefore not unreasonably be taken as a sign that his confidence in the authorities that overlook the work of that hospital was not a whit shaken by these attacks, and it is an example that the public would do well to follow.

The legacies to the various hospitals enumerated come at an opportune moment. The Royal Free has of late years spent large sums of money on absolutely necessary improvements, and there have been no further funds available for the much-needed extension of the out-patient department. All this expenditure has been required to bring the hospital up to the standard of modern demands, and has forced the committee to make the most strenuous efforts to avoid getting into debt. The work in the out-patient department at this hospital is conducted in a model fashion, for the Royal Free was the first London institution (and, we believe, also the first anywhere in the United Kingdom) to adopt the system of regular hospital almoners, thereby doing much to overcome the problem of hospital abuse. Mr. Silver's bequest will enable the authorities to deal with the out-patient extension in a prompt and energetic manner, since the money will form the nucleus of a building fund. Similarly the Great Ormond Street Hospital finds the windfall very acceptable at the present moment, when it is issuing a special appeal to meet a deficit of £7,000, which has to be paid off before Christmas. In the circumstances the appeal will be withdrawn, but we need hardly point out to friends of the institution that this hospital, with its great and increasing work among the children of the poor, is always deserving of the most cordial support. In the case of the Victoria Hospital at Chelsea many new improvements have lately been made, especially in the out-patient department, which has been remodelled. All this reconstruction work has cost money, and the bequest will prove a welcome addition to the ordinary funds of the institution.

It has been intimated to the secretaries of the three hospitals that benefit under Mr. Silver's will that the legacies due to them will, through the generosity of the residuary legatees, be paid out free of legacy duty. Thus the institutions concerned will get the actual sums as stated in the will, instead of having to pay to the Exchequer, in the case of the Royal Free, the sum of £5,000, and in the case of the two Children's Hospitals a sum of £2,500 each.

